# Ribosomal Database Project: data and tools for high throughput rRNA analysis

**DOI:** 10.1093/nar/gkt1244

**Published:** 2013-11-27

**Authors:** James R. Cole, Qiong Wang, Jordan A. Fish, Benli Chai, Donna M. McGarrell, Yanni Sun, C. Titus Brown, Andrea Porras-Alfaro, Cheryl R. Kuske, James M. Tiedje

**Affiliations:** ^1^Center for Microbial Ecology, Michigan State University, East Lansing, MI 48824, USA, ^2^Computer Science and Engineering, Michigan State University, East Lansing, MI 48824, USA, ^3^Microbiology and Molecular Genetics, Michigan State University, East Lansing, MI 48824, USA, ^4^Biological Sciences, Western Illinois University, Malcomb, IL 61455, USA and ^5^Bioscience Division, Los Alamos National Laboratory, Los Alamos, NM 87545, USA

## Abstract

Ribosomal Database Project (RDP; http://rdp.cme.msu.edu/) provides the research community with aligned and annotated rRNA gene sequence data, along with tools to allow researchers to analyze their own rRNA gene sequences in the RDP framework. RDP data and tools are utilized in fields as diverse as human health, microbial ecology, environmental microbiology, nucleic acid chemistry, taxonomy and phylogenetics. In addition to aligned and annotated collections of bacterial and archaeal small subunit rRNA genes, RDP now includes a collection of fungal large subunit rRNA genes. RDP tools, including Classifier and Aligner, have been updated to work with this new fungal collection. The use of high-throughput sequencing to characterize environmental microbial populations has exploded in the past several years, and as sequence technologies have improved, the sizes of environmental datasets have increased. With release 11, RDP is providing an expanded set of tools to facilitate analysis of high-throughput data, including both single-stranded and paired-end reads. In addition, most tools are now available as open source packages for download and local use by researchers with high-volume needs or who would like to develop custom analysis pipelines.

## INTRODUCTION

**Figure 1.**
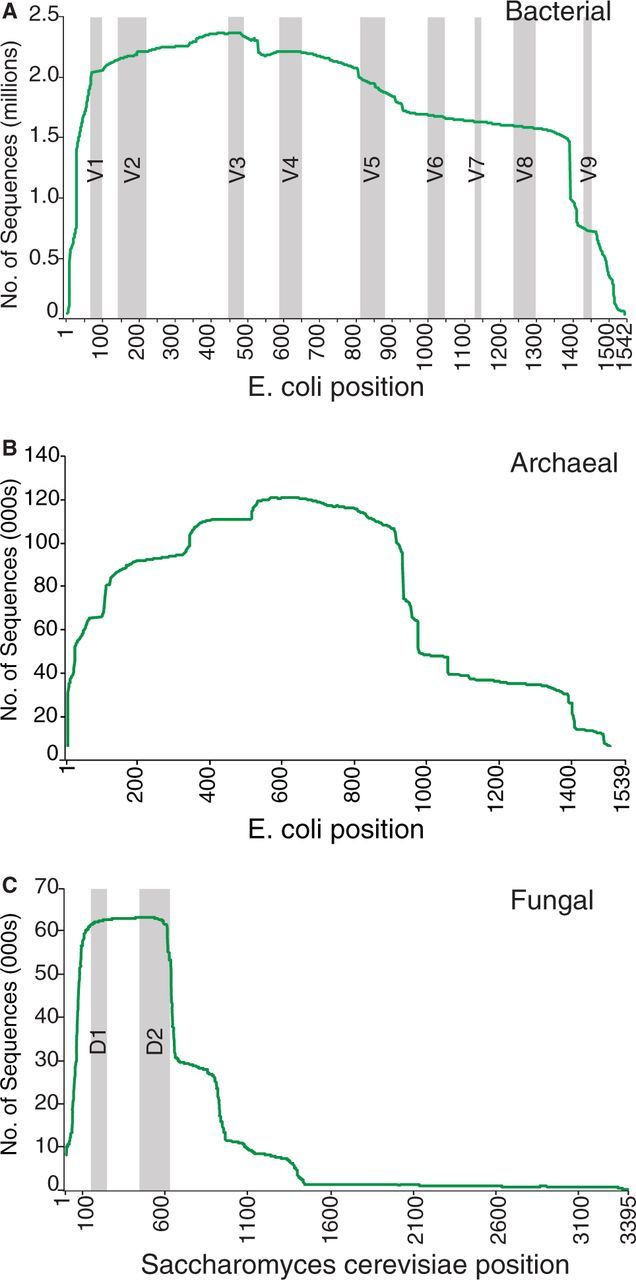
Gene coverage: number of sequences from RDP release 11.1 covering the indicated positions on the reference sequence. **(A)** Bacterial SSU rRNA gene. Positions relative to *Escherichia coli* sequence GenBank accession J01695.1. Gray bars indicate variable regions (1). **(B)** Archaeal SSU rRNA gene. Positions relative to *E. coli* sequence GenBank accession J01695.1. **(C)** Fungal LSU rRNA gene. Positions relative to *S. cerevisiae* GenBank accession NC_001144.5 LSU gene. D1 and D2 indicate hypervariable regions initially used for discrimination among *Fusarium* spp. (2). The D2 region is among the most highly variable eukaryotic LSU regions in terms of both length and structure (3). Such high diversity may improve the performance of the RDP Classifier when discriminating between closely related genera. Gene coverage charts are available online and updated with each incremental RDP release.

Ribosomal Database Project (RDP) 11.1, released in October 2013 (http://rdp.cme.msu.edu/), contains 2 809 406 aligned and annotated bacterial and archaeal small subunit (SSU) rRNA gene sequences and 62 860 fungal large subunit (LSU) rRNA gene sequences. The majority of rRNA gene sequences in the RDP database are incomplete. Most of these are derived from sequencing PCR amplification products, whereas a small number of older entries derive from reverse transcriptase sequencing of isolated rRNA. Because PCR amplification makes use of primers to conserved regions internal to the genes, few of these sequences cover the 3′ and 5′ ends of the genes (Figure 1). Still, a diverse selection of complete gene sequences, mostly derived from genome sequencing, is available. Only a relatively small percentage of bacterial and archaeal sequences originate from organisms in culture; roughly 85% and 97%, respectively, of bacterial and archaeal sequences in RDP are from DNA directly isolated from environmental samples.

Over the past several years we have been approached by a number of researchers interested in using RDP tools for analysis of fungi in the environment. With the latest release, we are providing both an alignment of fungal 28S rRNA gene sequences and a fungal training set for the RDP Classifier leveraging a recently published phylogenetically consistent taxonomic mapping (4). For our new fungal alignment, the number of sequences covering positions in the 5′ end of the gene is much higher than for the 3′ end (Figure 1C). The 28S gene is much longer than the bacterial 16S gene, and many fungal researchers appear to find that sequencing 5′ regions of the 28S gene provides sufficient phylogenetic resolution for strain differentiation.

RDP offers tools for browsing and searching the data collections, for taxonomic classification and nearest-neighbor search, for primer-probe testing and for tree building. In addition, RDPipeline tools are specifically designed for processing high-volume amplicon sequence data. New tools have been designed with speed and capacity in mind, and most previously published tools have been updated to accommodate the recent changes of the sequencing technology. Many RDP tools are also available as open-source stand-alone packages.

## RDP DATA COLLECTIONS

RDP obtains most of its rRNA sequences from the International Nucleotide Sequence Database Collaboration (INSDC; 5) databases. To prepare an RDP release, data files from the ‘standard’ dataclass and taxonomic divisions ‘Prokaryotes’, ‘Fungi’ and ‘Environmental Samples’ are downloaded from the European Nucleotide Archive (ENA; 6) ftp site. Records are examined for an ‘rRNA’ feature key of minimum length 500 bases (to allow sufficient context for taxonomic classification). If such record is found within an accession not in the RDP database, or within an existing accession but with a newer modification date, the sequence defined by the ‘rRNA’ feature is extracted. These new sequences are then filtered using a version of the RDP SeqMatch tool (described below) trained on a small hand-curated set of bacterial, archaeal, eukaryotic and mitochondrial SSU sequences and fungal LSU sequences. Only sequences having a best match to bacterial, archaeal or fungal with an S_ab score of at least 0.3 are saved. The original INSDC annotations, including structured comments such as Genomic Standards Consortium MIxS-compliant comments (7), are also captured. Many organism names in the INSDC records are not up to date. We obtain the most recent validly published synonym from Bacterial Nomenclature Up-to-Date (http://www.dsmz.de/).

Each sequence is aligned and classified using the corresponding RDP Aligner and Classifier (see below). Any sequence with a negative alignment bit saved score is discarded. The sequence is assigned to the lowest taxon in the RDP taxonomy with classification bootstrap confidence of 80% or above. Sequences passing this quality filtering are then subjected to the following tagging process. Sequences from type strains are tagged as ‘type’. Any sequence with accession listed in the bioproject.xml file (available from NCBI ftp site; 8) is tagged as a genome project sequence. All 16S rRNA gene sequences are screened for chimeric sequences using UCHIME (9) in reference mode. Positive UCHIME results are tagged as ‘suspect’ sequences. Next, the NCBI taxonomic assignment (10) is determined using the taxonomy ID in the db_xref qualifier obtained from the INSDC annotation. Any sequence assigned under an NCBI taxon with name containing ‘environmental’, ‘uncultured’ or ‘uncultivated’ is tagged as an ‘uncultured’ sequence. For each release, a set of flat files containing the entire sequence collection for each of the three genes are available for download in aligned or unaligned FASTA, and annotated GenBank formats. With each release, RDP provides the resource files for the NCBI LinkOut service. This allows researchers to jump directly to RDP sequence records from the corresponding records in the NCBI’s Nucleotide and BioProject databases (11).

### Alignments

The sequences in the RDP database are aligned using Infernal, a stochastic context-free grammar-based aligner (12). This aligner has several advantages: it incorporates secondary-structure information into the alignment process; as a model-based aligner, new sequences can be easily added to a pre-existing alignment; it is fast enough for very large numbers of sequences. The bacterial and archaeal aligners were trained using secondary structure information from the Comparative RNA Web Site (CRW; 13) and training alignments we developed with 2591 bacterial and 144 archaeal full-length sequences mostly from sequenced genomes, respectively. The bacterial training sequences have greater coverage (27 phyla) than those used for RDP release 10 (16 phyla). Many rRNA genes in genome sequences are annotated with incorrect start or stop positions. We adjusted these to produce consistent endpoints for the training set. We optimized the Infernal aligner parameters, particularly the relative entropy, to provide improved handling for partial sequences. Models and training sequences are available for download from the RDP website.

**Figure 2.**
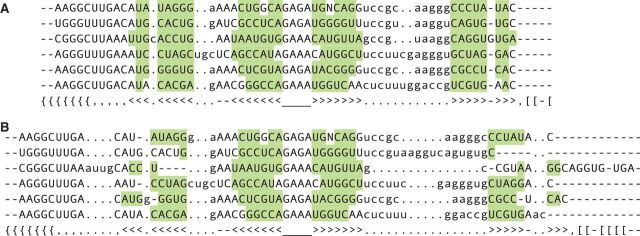
Multiple sequence alignment of partial bacterial 16S rRNA sequences corresponding to the region between common V6 variable region amplification primers (15). Uppercase columns correspond to modeled positions. Lowercase columns correspond to regions where hypervariability in size and structure preclude assignment of homologous residues. These columns are normally ‘masked out’ before phylogenetic analysis. **(A)** Using the new RDP 11 alignment model. This matches the alignment for this region obtained with full-length sequences. **(B)** Using the RDP 10 alignment model. The alignment of the full-length sequences is almost identical in this V6 region between the two models, except one G-U pair in RDP 11 appears as inserts in the RDP 10 alignment. Bases highlighted in green color are canonical base pairs matching the conserved secondary structure. From top to bottom, the GenBank accessions are AB006164, AB006178, AB021164, AB015577, AB003932 and AB004715.

The V6 region is especially hard to place into a multiple sequence alignment because much of the region is not conserved in size, sequence or secondary structure; however, the high diversity of the V6 region makes it a very common amplification target. Available tools often do not attempt to produce a multiple sequence alignment for amplicons of this region, but instead score pairwise alignments to a set of reference sequences for analysis (14). The tuned Infernal 1.1 aligner is able to correctly align the less hypervariable positions in the short region amplified by commonly used V6 primers (15), producing an alignment for this region matching that produced with full-length sequences (Figure 2).

We also tested this new alignment model by comparing the alignment produced using this model with the CRW bacterial seed alignment, which is hand-curated to match secondary structure. The Infernal alignment placed 92.7% of the bases in alignable positions (columns), the remainder corresponded to positions not conserved in the bacterial rRNA structure. We found that in 99.3% of the cases, pairs of residues in an alignable column of the Infernal alignment were mapped together to a column in the CRW alignment.

The fungal alignment uses a model built with 183 LSU sequences from complete fungal genomes plus the CRW fungal set, and covers four major fungal phyla: Ascomycota, Basidiomycota, Chytridiomycota and Blastocladiomycota. To develop the training model, we used a combination of the CRW general eukaryotic conservation model and *Saccharomyces cerevisiae* secondary structure model. In the large ribosomal subunit, the 5.8S and 28S molecules form a common secondary structure and the training model included the combined 5.8S and 28S gene sequences. (The Internal Transcribed Spacer ITS2 between 5.8S and 28S evolves too rapidly for global alignment and is treated as an insert in our model.) This fungal LSU Aligner is especially useful for aligning sequences resulting from protocols that amplify and sequence from all or part of the 5.8S gene to the 5′ portion of the 28S gene while not compromising alignment of sequences of only the 28S gene.

### Taxonomy

RDP bases its bacterial and archaeal taxonomies on the taxonomic roadmaps published by Bergey’s Trust (http://www.bergeys.org/outlines.html). As these are updated only at long intervals, we capture changes in taxonomic information and the publication of new species from the literature and from the List of Prokaryotic Names with Standing in Nomenclature website (16). We modify this taxonomy by adding clades for groups with few cultured relatives based on published informal taxonomies, where available. We compare this with the phylogenetic assessment from the All-Species Living Tree Project (17) and to our own assessment using the RDP Classifier. When there is a discrepancy between these sources, we conduct our own phylogenetic assessment by creating trees from the aligned sequences, including sequences from literature clades, and accept those clades best supported.

The fungal taxonomy used by RDP is the recently published taxonomy hand-developed using published phylogenies for different taxa and taxonomic databases (4) with updates. Because rRNA genes are too slowly evolving to reliably separate the validly named species (18), genus is the lowest rank presented in the RDP taxonomy. Where available, both genus and specific epithet, along with strain identifiers, are maintained for each sequence, but are not used to group or sort sequences. For species where our phylogenetically informed taxonomic assessment differs from the formal nomenclatural genus portion of the species binomial, the phylogenetically incorrect (but valid) name is maintained with the sequence and will differ from the assigned taxonomic lineage.

**Figure 3.**
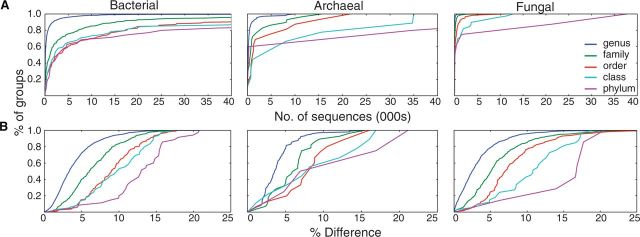
Accumulation curves showing **(A)** taxon size and **(B)** intra-taxon distance. All aligned sequences in RDP release 11.1 in each of the three RDP collections were clustered as described. The average distance between pairs of sequences in a taxon is shown in (B). The shape of the phylum curves, and to a lesser extent class curves, for archaea and fungi, are likely influenced by the small number of taxa and the skewed representation of sequences in these taxa.

Using the pairwise distances generated with the enhanced distance calculation tool included in the mcClust package (described below) and the sequences and taxonomic data available from the RDP database, we computed accumulation curves for the group size and intra-taxa distances at the genus, family, order, class and phylum level for each domain (Figure 3).

## TOOL DESCRIPTIONS

The RDP website provides an interactive interface to the RDP database and tools. RDP tools that accept sequence input from researchers do so via file upload or direct entry into a text field on the tool page. Recognized sequence file formats include FASTA, FASTQ, GenBank and EMBL formats. Most tools display results in a taxonomic hierarchy view that allows interactive browsing. Results are saved in session until the start of a new task, allowing researchers to switch between tools without losing their results. From most tools, RDP sequences can be selected and saved to a SeqCart, which in turn can be used by other RDP tools as input, or downloaded as aligned or unaligned sequence files, or as a distance matrix from the download page. Batch loading into the SeqCart is possible by uploading a file containing a list of INSDC accession numbers or RDP identifiers. Most tools are available as open-source command-line versions from the RDP GitHub repository (http://github.com/rdpstaff/).

### 

#### RDP Browsers

RDP Browsers provide interactive web interfaces to RDP’s sequence collections. The Hierarchy Browser enables researchers to navigate, search and select sequences from the RDP collections displayed in either the RDP or NCBI taxonomy. Data set options allow researchers to examine subsets of sequence records based on any combination of the following options: type or non-type strain sequences, uncultured or isolated organisms, partial or near-full-length sequences and suspect quality or good quality sequences. A search feature allows researchers to enter a word or words to be matched against sequence annotation. Advanced search features include Boolean logic, regular expressions and ability to limit search to a specified annotation field. The ‘display depth’ can be modified to control the number of ranks shown in the hierarchy. Researchers can add or remove sequences from their SeqCart by selecting individual sequences or entire taxa.

Two additional specialized browsers are available. The Genome Browser organizes rRNA sequences from genome projects and provides rRNA copy number and genome size plus link-outs to additional genome information hosted at other sites. Publication View organizes sequences by publications. Sequences for any individual publication can be displayed and selected in Hierarchy Browser.

*my*RDP is an account-based workspace that allows researchers to upload and store their pre-publication sequences. The facility is meant for single sequences up to groups of several hundred. These can be partial or complete rRNA sequences assembled from genomes or metagenomes, or sequences assembled from low-volume sequencing of rRNA gene clone libraries. The RDP Amplicon Sequence Pipeline (RDPipeline) described below is better suited for new amplicon sequencing technologies such as Illumina MiSeq. Sequences are uploaded in sequence groups to *my*RDP, and these groupings are maintained. Upon uploading, sequences are automatically submitted for alignment and classification. These *my*RDP sequences can then be analyzed in combination with sequences from RDP’s collections using RDP’s tool suite. A special social network feature allows sequence groups to be shared with additional researchers (‘research buddies’) specified by sequence owners. This feature is especially useful for remote collaborations.

#### Sequence Match (SeqMatch)

This is one of the most often used online RDP tools. It is a re-implementation of the original RDP SIMILARITY_RANK (19,20). SeqMatch finds closest RDP sequences to a query based on the fraction of shared seven-base sequence fragments (words) between the query and reference sequences (S_ab score). SeqMatch works well on partial- and full-length sequences and is more accurate than BLAST (21) at identifying database sequences that are closely related to query rRNA sequences.

The online SeqMatch is a *k*-nearest neighbor classifier and displays each query sequence under the lowest common ancestor taxon consistent with the *k* top matches for that query. In Detail View, these top *k* matches are all presented in a taxonomic hierarchy display similar to the Hierarchy Browser.

The standalone SeqMatch is available from the RDP GitHub repository. It requires an input sequence file, a reference sequence file and optional S_ab cutoff and *k* value. The output file contains the following information for each *k* top matches to a query: query name, match sequence ID, orientation, S_ab score and the number of unique common 7-mers.

#### Classifier

The RDP Classifier rapidly and accurately assigns sequences into taxa with bootstrap value, an estimate of confidence for each assignment (22). The RDP Classifier has several advantages over most other methods of classifying rRNA sequences, especially for large high-throughput sequencing datasets: high speed with minimum memory requirement, does not require alignment, works well for partial sequences and can be easily retrained with alternative taxonomy or for different genes. The online RDP Classifier is pre-trained for bacterial and archaeal 16S and for fungal 28S rRNA gene sequences (see ‘Taxonomy’ for more detail). The bacterial and archaeal 16S training set has been updated seven times since the first release to reflect changes in taxonomic opinions. The online tool takes input query sequences and a choice of training set. The results are shown in a taxonomic hierarchy view displaying all the taxon nodes with sequences assigned to them. Researcher can change the ‘confidence threshold’ to choose a cutoff suitable for the dataset. For partial sequences, using a lower confidence cutoff has been shown to increase the classification coverage at genus rank with sufficient accuracy (23). A detailed view shows individual queries assigned to each taxon.

The current version of the Classifier incorporates a number of enhancements not covered in the initial publication. The bootstrap assignment strategy has been changed to avoid an over-prediction problem when multiple genera are tied for highest score during bootstrap trials. The Classifier now allows multiple sample inputs. Expanded output options include detailed classification assignment for each sequence and an output file with one column for each sample containing assignment counts for each taxon. The latter is in a format appropriate for beta diversity analysis and sample ordination, and produces results similar to those obtained from operational taxonomic unit (OTU) clustering-based methods (24).

The command-line Classifier (available from the RDP GitHub repository) provides extensive support for retraining, allowing researchers to rapidly test the consistency of their training sets and to flag possible errors in their custom taxonomies. There is no requirement for a uniform number of taxonomic ranks and uncommon ranks are correctly handled. The classification speed is proportional to the number of genera, not the number of training sequences. This allows custom training sets with very large numbers of sequences. However, a larger training set with less accurate assignments or taxonomic irregularities will not necessarily work well—the testing tool can help validate a new training set. These features have allowed researchers to retrain the RDP Classifier on a broader range of sequences, including ones from environmental clades (25), on honeybee gut specific 16S rRNA sequences (26) and on fungal LSU sequences (4).

#### Library Compare

RDP Library Compare (22) is used to investigate statistical differences between a pair of sample libraries. Instead of estimating overall difference between samples, LibCompare provides *P* values for determining statistical significance of abundance differences for individual taxa. This tool first uses the RDP Classifier to assign sequences to taxa. Depending on the abundance of sequences assigned to each taxon, one of two statistical tests is used to compute a *P* value to determine if a taxon is differentially represented in the two libraries.

The standalone Library Compare is available as part of the RDP Classifier package. It produces an output containing assignment detail result for each query, and a tab-delimited file containing comparison results sorted by *P* value. Each line contains the *P* value, taxon rank, taxon name and the number of assignments from each sample.

#### Probe Match

Probe Match (20) performs a search against a sequence dataset for matches to the entered oligonucleotide sequences (primer). This tool implements a fast bit-vector algorithm for approximate substring matching (27). The online Probe Match takes primer sequences in Standard IUPAC code (allowing degenerate bases). There is an option to check a pair of primers in tandem, effectively testing *in silico* PCR. Researchers choose which of the three RDP sequence collections to search: Bacteria, Archaea or Fungi. Researchers can also limit the search to only the sequences containing a specified region of the molecule. This does not limit the search to that region, but by removing partial sequences missing the expected target site, it gives a more accurate estimate of primer coverage.

The standalone Probe Match general-purpose search engine (available from the RDP GitHub repository) requires an input sequence file and one or more primer sequences. Input sequences can be from any genes and of any length, but primers must not be longer than 64 characters. Multiple primers can be used at the same time, but for each sequence only the result for the best matching primer is reported. The output file contains sequence IDs that match at least one of the primer(s) within the specified distance and the detailed information of the match.

#### RDP Aligner

RDP offers two ways for researchers to align sequences. Any bacterial or archaeal 16S gene sequences uploaded to a researcher’s *my*RDP account are automatically aligned. Researchers can also align bacterial and archaeal 16S as well as fungal 28S sequences using the Aligner on the RDPipeline website. The online Aligner uses the same Infernal alignment models used to process the RDP database sequences (see ‘Alignment’ section above). The Aligner has been updated to Infernal version 1.1. This version is 7.5 times faster than the previous version used with RDP release 10. Since the standalone Infernal does not check the orientation of sequences; the online RDP Aligner first checks the orientation of each sequence and reverse-complements if necessary before aligning. The RDPipeline contains a suite of tools for further processing aligned sequence sets (see below). The Infernal 1.1 models used in RDP release 11, as well as the Infernal 0.81 models used in RDP release 10 are available from the RDP GitHub repository.

#### Tree Builder

Tree Builder constructs a phylogenetic tree from sequences selected from the RDP collections and *my*RDP in any combination. This tool uses the Weighbor (28) weighted neighbor joining method with Jukes–Cantor corrected distances calculated from the RDP alignment. Bootstrap confidence estimates for each tree node are calculated from 100 bootstrap resamplings of the alignment columns. Normally an outgroup sequence should be included to allow the tree to be correctly rooted. The resulting tree is displayed in a Java applet that allows interactive exploratory manipulations, such as selecting nodes, and swapping branches. The trees can be downloaded in standard Newick format as well as in PS/PDF file formats.

#### Assignment Generator

The Assignment Generator provides support for a 16S rRNA gene analysis lesson plan (29). It introduces comparative 16S rRNA gene analysis through a realistic bioinformatics exercise (unique sequences, common research tasks) that is easy to manage, distribute and evaluate using tools on RDP’s website. An instructor can generate a set of unique assignments for the entire class by specifying the number of students in the class, the number of sequences for each student and the choice of dataset (among bacteria, bacteria and archaea, or medically important bacteria). The tool provides: (i) a unique set of sequences for each student that are derived from the RDP sequence collections in a way that preserves secondary structure, (ii) a set of directions describing assignments for the students and (iii) an evaluation key for the instructor with the expected results for each student. This tool has been used in classes of sizes up to more than 500 students.

### RDPipeline for high-throughput amplicon analysis

The RDPipeline performs several common processing steps in taxonomy-dependent (using the RDP Classifier), and taxonomy-independent (using hierarchical clustering) analysis of large datasets. The RDPipeline is a new tool suite designed to replace our previous Pyrosequencing Pipeline (30), offering extended processing and analysis tools reflecting recent shifts in amplicon sequencing technologies and techniques. Researchers can utilize the tools in the RDPipeline tool suite in one of two ways. For researchers processing a moderate amount of sequences, we offer online versions of the RDPipeline tools. For researchers involved in high-volume sequencing projects, or who would like to incorporate some of our tools into their local custom workflow, we offer all the tools that make up the RDPipeline on the RDP GitHub repository.

The online RDPipeline has integrated support for *my*RDP accounts. All submitted jobs are viewable from the ‘my jobs’ page. Analysis results are stored for up to 2 weeks but job history remains available. The job history lists the type of each job, current status, submission, start and completion times and supplied processing parameters. For long running jobs, an email containing a direct link to download the results is sent when processing has completed. All RDPipeline tools accept compressed files. Any compressed file will be expanded upon upload and the contained files are treated as the input to the tool. All RDPipeline tools have extended input validation checks on uploaded files. Before processing begins, a summary is displayed showing the files detected and any files not used because of unexpected file type.

**Figure 4.**
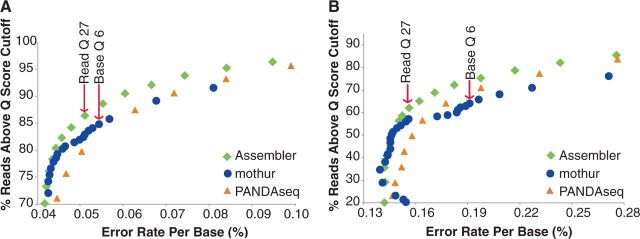
Comparing per base error rates for three paired-end read assembly tools. The error rates were calculated using assembled reads filtered by either read *Q* score (Assembler and original PANDAseq; 38) or delta *Q* score (mothur; 39). Recommended read *Q* score of 27 for Assembler and base *Q* score (deltaq) of 6 for mothur are marked. **(A)** Sample M_20130714 and **(B)** Sample M_20130819.

‘Initial processing’ prepares raw sequences from a sequencing facility for analysis. It is a multi-step process that includes sorting the raw reads by sample tag, trimming off tag and primer regions and removing sequences of low quality. Input file can be a single file or a compressed file containing multiple sequence files. For paired-end data, it uses our Assembler (described below; Figure 4) to assemble overlapping paired reads as the first step. We recommend researchers analyzing paired-end data use a read *Q* score cutoff of around 25–27 to filter out low-quality assembled reads. Summary statistics are included in the download for each tag, including the number of sequences filtered by each filter and a histogram of sequence lengths after filtering.

#### Alignment

This tool allows researchers to align up to 1 000 000 Bacterial/Archaeal 16S or Fungal 28S sequences at a time with Infernal 1.1 using the RDP alignment models. Uploaded sequences for all genes are checked for orientation and reverse complemented when necessary. Each alignment job result also includes alignment position and length statistics as well as a summary histogram of read alignment start and end positions relative to the alignment model.

#### Clustering

The complete linkage clustering tool (29,30) allows users to upload aligned sequences to be clustered as the first step in taxonomy-independent analysis. Sequence files can be clustered together with each file treated as a sample, or files can be clustered separately. The online clustering tool is limited to 150 000 unique sequences per job. For clustering very large datasets, we provide a modified version of mcClust (31) for download (see below). This new version distributes distance calculations among a compute cluster and incorporates algorithmic changes that lower the time complexity and speed up clustering.

#### Ecological measures

The cluster file obtained from Clustering or mcClust can be used to compute five common ecological measures for their samples. Alpha diversity can be estimated using Shannon or Chao1 and beta diversity can be measured using the Jaccard or Sørensen indices. Researchers can also assess sequencing depth using the rarefaction tool.

#### Sequencer run quality checks

The RDPipeline includes two tools, Defined Community Analysis and Chimera Check for assessing the quality of sequence runs (31). The latter is powered by UCHIME (9). For researchers who include a defined community sample in their sequencer run, the Defined Community Analysis Tool calculates the observed error rates based on the known gene sequences of the organisms in the defined community.

#### Additional tools

Researchers can use the ‘Cluster File Format Conversion’ tool to convert RDP Cluster files to an OTU table format, suitable for input to R and estimateS, or to the BIOM format (32). The ‘Alignment Merger’ tool allows researchers to merge alignment files created independently. The ‘Sequence Selector’ tool allows researchers to upload a set of sequence files and a separate file containing a list of IDs. A file is returned either containing only the sequences specified, or excluding them, depending on option selected. The ‘Representative Sequence Selector’ tool allows researchers to upload a cluster file and retrieve a ‘representative sequence’ from each cluster, defined as the sequence with least sum of squared distances to all other sequences in the cluster.

#### mcClust enhancements

Hierarchical sequence clustering methods that worked well for thousands of amplicon sequences often fail with the increased output of the latest sequencing technologies. Exact clustering methods require all pairwise distances for the input sequences and thus scale on the order of O(*n*^2^). Many clustering implementations, in addition to requiring O(*n*^2^) computational time, also have a memory complexity of O(*n*^2^) as they store all distances in memory. Nonetheless, clustering methods remain an important tool in rRNA sequence analyses, and several groups have attempted to solve the scaling issues facing sequence clustering. One approach is to adopt an approximate clustering method, as in USEARCH (33) and CD-HIT (34), which use heuristics to limit the number of pairwise comparisons calculated. Another approach proposed by Loewenstein *et al.* (35) focuses on limiting the memory complexity of average linkage clustering by storing the distances on disk. To utilize disk storage for the pairwise distances, they must be in sorted order. With a general purpose sorting algorithm this increases the time complexity to O(*n*^2^log *n*^2^).

Several previously published complete linkage algorithm implementations take advantage of on disk storage of distances to limit the memory requirements for clustering (31,36,37). These implementations still require all pairwise distances (or at least all pairwise distances up to a maximum distance cutoff), and more importantly require sorting of all these distances. We propose a distance calculation tool with the goal of being efficient in the distance calculations, allowing the distance matrix computation to be parallelized and using an alternative sorting method to reduce the time complexity back to O(*n*^2^) (see Supplementary material).

The distance calculation tool is implemented in Java 1.6 and has been integrated as a tool into the mcClust package (31) available on the RDP GitHub repository.

#### Assembler for paired-end reads

Compared to single-stranded Illumina reads, assembled paired-end reads can provide longer sequences with lower error rates. However, newly developed paired-end assembly tools have limitations. We have extended the existing PANDAseq (38) paired-end reads assembly program. Our modified PANDAseq (Assembler) performs a modified statistical analysis using the sequencer supplied quality (*Q*) scores to find the most likely overlap, computes assembled *Q* scores for the read overlap region and handles more complex overlap layouts (see Supplementary material for details).

We have tested Assembler using two defined community samples from two different MiSeq runs. Both runs passed the Illumina MiSeq quality standards but the basic per base error rates of these two samples are quite different (0.17% for sample M_20130714 and 0.7% for sample M_20130819 after assembly). Both are within the reported error rate range for paired-end MiSeq amplicon data (0.28–1.08%) (39). Using an overall read *Q* score quality filter to remove low quality sequences, we tested the Assembler against the paired-end assembler and quality filter built into mothur (39), another amplicon analysis program. Assembler slightly outperformed mothur on the high-quality dataset (Figure 4A), and significantly outperformed on the average-quality dataset (Figure 4B). In both datasets, Assembler outperformed the original PANDAseq when scored in a similar manner (although such *Q* score based filtering was not a goal of that implementation). Using a read *Q* score of 27 decreases the error rates to 0.05% and 0.16% for M_20130714 and M_20130819, respectively, and was effective in selectively removing reads with a high number of errors (Supplementary Figure S3). The Assembler is integrated into Initial Processing and is available on the RDP GitHub repository.

All three programs can be run with multiple threads but were limited to a single thread in our testing. On an AMD Opteron 8384 quad-core 2.7 GHz processor, it took Assembler 1.4 h to assemble over 16 million reads from one MiSeq run. The original PANDAseq took 20 min, while mothur took 21.3 h to assemble the same set of data using its recommended analysis protocol, on the same system.

## USER SUPPORT

RDP’s mission includes user support. RDP online tools are each supplied with a help page as a quick reference for its functionality, algorithm and how-tos. An RDP Wiki provides an updated searchable repository for answers to commonly asked questions compiled from previous user communications with RDP staff. Workflow tutorials guide researchers through common task-oriented processes, provide sample data and introduce researchers to the best practices for NGS data analysis. For command-line tools, step-by-step instructions and sample data files are provided on the RDP GitHub repository. Support questions can be emailed to rdpstaff@msu.edu. Telephone support is available (+1 517 432 4998).

## AVAILABILITY OF SUPPORTING DATA

The sequence data from this study have been submitted to the ENA Short Read Archive (http://www.ebi.ac.uk/ena/) under accession no. PRJEB4878.

## SUPPLEMENTARY DATA

Supplementary Data are available at NAR Online.

## FUNDING

Office of Science (Biological and Environmental Research), US Department of Energy [DE-FG02-99ER62848]. Additional support came from the Office of Science (Biological and Environmental Research), US Department of Energy [DE-SC0004601] and Bioenergy Center [DE-FC02-07ER64494]; the US National Institute of Environmental Health Sciences Superfund Research Program [P42 ES004911]; the National Science Foundation [DBI-0328255]; the US Department of Agriculture National Institute of Food and Agriculture National Research Initiative [2008-35107-04542]; the National Institute of Health Research Project [U01 HL098961] and Human Microbiome Project Demonstration Project [UH3 DK083993]. Funding for open access charge: US Department of Energy.

*Conflict of interest statement.* None declared.
